# Racial and Sex Differences in 24 Hour Urinary Hydration Markers among Male and Female Emerging Adults: A Pilot Study

**DOI:** 10.3390/nu12041068

**Published:** 2020-04-12

**Authors:** William M. Adams, Derek J. Hevel, Jaclyn P. Maher, Jared T. McGuirt

**Affiliations:** 1 Department of Kinesiology, University of North Carolina at Greensboro, Greensboro, NC 27412, USA; djhevel@uncg.edu (D.J.H.); jpmaher@uncg.edu (J.P.M.); 2 Department of Nutrition, University of North Carolina at Greensboro, Greensboro, NC 27412, USA; jtmcguir@uncg.edu

**Keywords:** fluid intake, urine osmolality, urine volume, urine specific gravity, water consumption

## Abstract

The purpose of this study was to examine 24 h urinary hydration markers in non-Hispanic White (WH) and non-Hispanic Black (BL) males and females. Thirteen males (BL, *n* = 6; WH, *n* = 7) and nineteen females (BL, *n* = 16, WH, *n* = 3) (mean ± SD; age, 20 ± 4 y; height, 169.2 ± 12.2 cm; body mass, 71.3 ± 12.2 kg; body fat, 20.8 ± 9.7%) provided a 24 h urine sample across 7 (*n* = 13) or 3 (*n* = 19) consecutive days (148 d total) for assessment of urine volume (U_VOL_), urine osmolality (U_OSM_), urine specific gravity (U_SG_), and urine color (U_COL_). U_VOL_ was significantly lower in BL (0.85 ± 0.43 L) compared to WH college students (2.03 ± 0.70 L) (*p* < 0.001). Measures of U_OSM_, U_SG_, and U_COL_, were significantly greater in BL (716 ± 263 mOsm∙kg^−1^, 1.020 ± 0.007, and 4.2 ± 1.4, respectively) compared to WH college students (473 ± 194 mOsm∙kg^−1^, 1.013 ± 0.006, 3.0 ± 1.2, and respectively) (*p* < 0.05). Differences in 24 h urinary hydration measures were not significantly different between males and females (*p* > 0.05) or between the interaction of sex and race/ethnicity (*p* > 0.05). Non-Hispanic Black men and women were inadequately hydrated compared to their non-Hispanic White counterparts. Our findings suggest that development of targeted strategies to improve habitual fluid intake and potentially overall health are needed.

## 1. Introduction

Water is an essential component for life and is intimately involved in maintaining normal physiologic function throughout the body. The complex and dynamic processes of total body water turnover and fluid regulation vary from person to person making it difficult to define a universal standard for daily adequate water intake [1,2,3]. However, entities such as the European Food Safety Authority (EFSA) [4] have developed adequate intake values of water for men (2.5 L/day) and women (2.0 L/day), which were derived from data assessing daily water consumption and a theoretical value for urine osmolality.

Recent epidemiological data has suggested a possible link between the volume of daily water intake and various health outcome measures. Increased consumption of water has been associated with a reduced risk of obesity, urinary tract infections, chronic kidney disease, cardiovascular disease, and a reduction in the incidence of hyperglycemia [5,6,7,8,9,10,11]. Despite these benefits, recent cross-sectional surveys have shown that 60% of men and 40% of women do not comply with the EFSA recommendations for daily water intake [12], which may alter the long-term health risk profile in persons who habitually consume low volumes of water on a daily basis [13,14]. Although 40% of women were found to not meet current EFSA standards, they were more than twice as likely to meet the adequate intake standards for daily fluid intake than that of men [12]. 

Furthermore, studies suggest that there are racial and ethnic differences surrounding fluid intake with non-Hispanic Blacks being inadequately hydrated compared to non-Hispanic Whites [15,16,17]. Kenney et al. [16], found that children who were non-Hispanic Black and/or a boy were more likely to be underhydrated than non-Hispanic Whites and girls, respectively. Similarly, Brooks et al. [15] observed that non-Hispanic Black adults were 1.4 times more likely than non-Hispanic White adults to be inadequately hydrated as measured by urine osmolality and assessment of total water intake in the participants’ diet. Notwithstanding, in addition to racial/ethnic differences in water intake, factors such as age, sex, and level of physical activity may also contribute to observed differences [17]. 

However, these previous studies have been limited with regard to various aspects of the methodological approach [18,19], which may inaccurately depict true racial and sex differences in daily hydration status. Specifically, there are two key limitations of this previous research. First, previous findings only utilized a spot urine sample, which is subject to circadian variation [20,21,22] and has been shown not to reflect 24 h hydration status. Moreover, 24 h urine osmolality has been proposed as measure to determine optimal hydration status as it provides an accurate assessment of total fluid intake [23]. By not utilizing a clinically validated method for assessing daily fluid intake and 24 h hydration status, previous results may not accurately depict a true assessment of daily hydration status across multiple days [24,25,26]. Second, while previous literature has largely focused on explaining hydration differences in a given population [27,28], there is no known literature that has specifically looked at racial and sex differences in 24 h hydration status in emerging adults (18–25 y). 

Given the aforementioned limitations, gaining a better understanding of daily hydration status in emerging adults is needed. Emerging adulthood represents a critical time in life due to the transition from dependency on a caregiver to independence, which may result in the adoption of poor health behaviors. Therefore, the primary purpose of this study was to examine the racial and sex differences in 24 h urinary hydration measures in non-Hispanic Black and non-Hispanic White emerging adults. As a secondary aim, we sought to explore the interactions between sex and race on 24 h urinary hydration measures in this population.

## 2. Methods

### 2.1. Participants

Thirteen men (non-Hispanic Black (BL), *n* = 6; non-Hispanic White (WH), *n* = 7) and nineteen women (BL, *n* = 16, WH, *n* = 3) (mean ± SD; age, 20 ± 4y; height, 169.2 ± 12.2 cm; body mass, 71.3 ± 12.2 kg; body fat, 20.8 ± 9.7%) (see Table 1 for further participant characteristics) were recruited from a university in the southeastern United States to participate. Participants were excluded if they met any of the following exclusionary criteria: (1) evidence of clinically relevant diseases that may alter body water regulation, (2) previous surgery on the digestive tract that may impair the body’s ability to normally regulate body water, and (3) actively attempting to gain or lose body weight, which may influence total water intake due to changes in caloric intake. In addition, female participants were excluded if they were currently pregnant. To assess race/ethnicity, all subjects that self-identified as non-Hispanic White or non-Hispanic Black were included. All participants were informed of the study requirements, benefits, and risks before providing written informed consent. This study was approved by the University of North Carolina at Greensboro’s institutional review board (approval numbers, 18-0269 and 18-0063) prior to the commencement of any data collection.

### 2.2. Procedures

This study combined results from two separate studies that utilized an observational design where participants visited the laboratory for 7 (*n* = 13) or 3 (*n* = 19) consecutive days for 24 h hydration assessment. In total, 148 d (observations) of 24 h urine samples were collected amongst all participants for all days they were instructed to come to the laboratory. The study requiring participants to visit the laboratory on 3 consecutive days to drop off 24 h hydration assessment included one weekend day and two weekdays. During the initial visit to the laboratory, a trained research assistant took anthropometric measures and participants were instructed on the 24 h data collection procedures. Participants were provided with a clean container in which they were to void all urine produced over a 24 h timeframe. Participants were instructed to provide a complete urine void for each time point. For male participants, they were instructed to void directly into the 10 cm diameter opening of the clean container. For female participants, they were provided a graduated specimen pan (Model DYND36600, Medline Industries, Inc., Northfield, IL, USA) to void all urine. Following each void, female participants were instructed to pour the void into the clean container provided to them in order to seal and store the sample. Participants arrived the next morning to the laboratory, returned their 24 h urine sample and provided a nude body mass. Participants were given a clean specimen container and were instructed to repeat the same procedures daily for the length of the study.

To minimize individual variability and the influence of circadian rhythms in hydration status, all participants arrived at the laboratory to return urine collected in the previous 24 h between the hours of 0600–0900 each day with the timing of their arrival being ±1 h from their initial visit. Participants were instructed to go about their normal daily routines during the urine collection periods to garner an ecologically valid assessment of their 24 h hydration status. 

### 2.3. Measurements

Hydration Status. For assessment of hydration status, each 24 h urine sample was measured for urine volume (U_VOL_) using a digital scale to the nearest 0.0001 kg (Ranger 3000, OHAUS Corporation, Parsippany, NY), urine osmolality (U_OSM_) performed in duplicate using freezing point depression (Model 3320, Advanced Instruments, Inc., Norwood, MA), urine specific gravity (U_SG_) (Reichert AR200, Reichert Technologies, Buffalo, NY), and urine color (U_COL_) [29]. For the purposes of this manuscript, euhydration was defined as a U_OSM_ < 500 mOsm∙kg^−1^ [23], U_SG_ ≤ 1.012, and U_COL_ ≤ 4 [30].

Body Composition. Nude body mass was measured to the nearest 0.1 kg using a digital scale (WB-800S Plus, Tanita Corporation, Tokyo, Japan) and height was measured to the nearest 0.1 cm using a wall-mounted stadiometer (Model 216, Seca, Chino, CA, USA). Body density was measured using the methods established by Jackson and Pollock [28,29]. The average of two skinfold measures (Lange Skinfold Calipers, Beta Technologies, Santa Cruz, CA) taken across three sites on the right side of the body were used; measures were taken at the chest, abdomen and thigh for men [31] and suprailiac, triceps, and thigh for women [32]. Body fat percentage was estimated using the method established by Siri [33].

### 2.4. Statistical Analysis

All data analyses were performed using SPSS (v. 24.0, IBM Corporation Armonk, NY, USA). All data are presented as mean ± SD unless otherwise noted. Differences in hydration marker variables between independent variables are presented as mean difference (MD) and 95% confidence intervals [CI]. Intra-individual means were calculated for each independent variable. Data were grand mean centered and differences in 24 h hydration status between race/(non-Hispanic black/non-Hispanic white) and sex (male/female) were assessed using repeated measures linear mixed effects models with race and sex as fixed factors. Bonferroni post hoc analyses were conducted when appropriate. Significance was set a-priori at *p* < 0.05.

Post hoc power analysis comparing 24 h urine volume between WH and BL participants revealed that this study was sufficiently powered to detect an effect size of 2.14; using an alpha level of *p* = 0.05, and beta level at β = 0.80, the achieved power was β = 0.84.

## 3. Results

Participant characteristics are shown in Table 1. Across 148 days, participants were classified as underhydrated 54.7% (*n* = 81 d), 29.7% (*n* = 44 d), and 20.9% (*n* = 31 d) when using previously defined thresholds for U_OSM_ (>500 mOsm∙kg^−1^), U_SG_ (>1.012), and U_COL_ (>4), respectively. Furthermore, 24 h U_VOL_ exceeded 1.0 L, 1.5 L, and 2.0 L on 62.2% (*n* = 92 d), 46.6% (*n* = 69 d), and 25.0% (*n* = 37 d), respectively. No significant differences were observed in U_VOL_ (*p* = 0.113), U_OSM_ (*p* = 0.108), U_SG_ (*p* = 0.166), and U_COL_ (*p* = 0.466) between weekdays and weekend days (Table 2)

**Figure 1:**
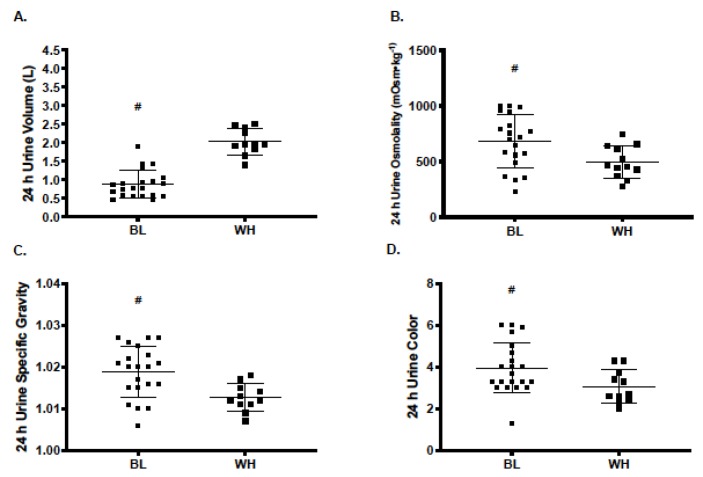
Differences in (**A**) 24-h urine volume, (**B**) 24-h urine osmolality, (**C**) urine specific gravity, and (**D**) urine color between non-Hispanic Black and non-Hispanic White college-aged males and females. # indicates statistical significance (*p* < 0.01).

Difference in 24 h hydration variables between BL and WH male and female college students are shown in Table 3. U_VOL_ was significantly lower in BL compared to WH college students (MD [95% CI]; −1.28 L [−1.57, −0.99], *p* < 0.001). Conversely, measures of U_OSM_ (246 mOsm∙kg^−1^ [101, 390], *p* = 0.001), U_SG_ (0.007 [0.004, 0.011], *p* < 0.001), and U_COL_ (1.3 [0.5, 2.1], *p* = 0.002), were significantly greater in BL compared to WH college students (Figure 1).

There were no significant differences in 24 h urinary hydration measures (U_VOL_, *p* = 0.397; U_OSM_, *p* = 0.942; U_SG_, *p* = 0.667; U_COL_, *p* = 0.249) between males and females (Table 3). Similarly, there were no significant interactions between race/ethnicity and sex for U_VOL_ (*p* = 0.232), U_OSM_ (*p* = 0.571), U_SG_ (*p* = 0.550), or U_COL_ (*p* = 0.505). 

## 4. Discussion

This is the first known study that has examined racial and sex differences in 24 h hydration status in emerging adults. Our findings show that non-Hispanic Black college students, regardless of sex, are inadequately hydrated compared to non-Hispanic white college students when assessing 24 h urine samples for total volume, osmolality, specific gravity, and color. We found no significant differences between males and females, nor any significant interactions in 24 h urinary hydration measures between sex and race.

The current study found non-Hispanic Whites to be more hydrated than non-Hispanic Blacks and was confirmed by 24 h urine volume (2.03 vs. 0.85 L), urine osmolality (473 vs. 716 mOsm∙kg^−1^), urine specific gravity (1.013 vs. 1.020), and urine color (3.0 vs. 4.2). Furthermore, we found that non-Hispanic Blacks were underhydrated, defined by a urine osmolality greater than 500 mOsm∙kg^−1^ [29], on 77.1% (54/70) of days compared to 34.6% (27/78) of days for the non-Hispanic White participants in this study. Given prior literature (23), utilizing a 24 h urine osmolality <500 mOsm∙kg^−1^ as a threshold of optimal hydration, and increased likelihood of an individual meeting daily adequate intake volumes of water as established by EFSA, we can estimate that 22.9% of non-Hispanic Blacks and 65.4% of non-Hispanic Whites in our study were meeting daily water intake requirements. Our findings are in line with others who have found that non-Hispanic Blacks were inadequately hydrated compared to non-Hispanic Whites in a large population based sample [15,16,17]. While we did not explore reasons that contributed to inter- and intra-individual decisions surrounding daily fluid intake, we speculate that prior life experiences related to knowledge of hydration on health, perceptions of safe drinking water, and availability and accessibility of safe and affordable sources of fluids may have influenced our participants daily fluid consumption as measured by their 24 h urine sample as suggested in prior work [17,25,34,35]. 

Other considerations that must not be discounted for potential differences in 24-h urinary hydration measures are the differences in dietary patterns observed between non-Hispanic Black and non-Hispanic White populations [36,37,38]. Deshmukh-Taskar et al. [36], found that non-Hispanic Blacks consumed more servings of a ‘Western Dietary Pattern’, reflective of foods that contain less water content than a ‘Prudent Dietary Pattern’, which includes high water content foods such as fruits and vegetables. As approximately 20%–25% of an individual’s daily fluid intake comes from food intake [39], differences in dietary patterns may contribute to observed differences in 24 h urinary hydration measures, however, our current pilot study did not assess for this.

While our study results align with the aforementioned work [15,16,34], our study may be more reflective of one’s daily hydration status because we used a 24 h urine sample across multiple consecutive days for hydration assessment. These methods allow for the capture of individual and daily variation in 24 h hydration status [21,22,40,41,42], as well as providing greater clinical utility when guiding recommendations for individual fluid intake for long term health. Interestingly, our findings also show that, within this sample population of college students, there were no differences in 24 h urinary hydration measures when comparing weekdays and weekend days (Table 2). These findings suggest that 24 h urinary hydration variables are relatively stable across the week despite potential differences in the structure of weekdays (e.g., class, studying, other school-related responsibilities) compared to weekend days (e.g., employment responsibilities, increased recreational and social activities, etc.).

Our study also found that that there were no differences in 24 h urine volume, urine osmolality, urine specific gravity and urine color between males and females. Specifically, we found that males and females were hypohydrated (urine osmolality >500 mOsm∙kg^−1^) 50% and 60.6% of the time, respectively. This is in contrast to prior work [43,44] that found men to have a higher urine osmolality than women. A potential reason for these contrasting findings is the study populations between studies; our study enrolled non-Hispanic White and non-Hispanic Black college students whereas the aforementioned work studied older Portuguese men and women. Given the observed geographical differences in habitual fluid intake across the lifespan [27,28] and the known changes in habitual fluid intake as one ages [26,45,46,47,48], the generalizability of previous work to the current study may be limited. 

Another explanation to account for the lack of observed differences in 24 h urinary hydration status when comparing sex and the interaction of sex and race is the distribution in sample size in the current study, particularly with the number of female participants. Nineteen non-Hispanic Black females completed the current study compared to the three non-Hispanic White females. Given the statistically significant differences in 24 h hydration status based on race/ethnicity in our study, the unequal sample sizes may have prevented us from elucidating the influence of sex and ethnicity on 24 h urinary hydration measures.

The strengths of the current study are the utilization of 24 h urine samples across consecutive days to examine inter- and intra-individual differences in daily hydration status. These measures are informative surrounding daily fluid intake and whether individuals are meeting daily intake recommendations. By assessing 24 h urinary hydration variables, we are able to minimize the influence of sex and race/ethnicity and the systematic variation in urinary flow rate as observed in assessment of spot samples [49], thus providing a more accurate depiction of racial and sex differences in urinary hydration variables. Furthermore, given the differences observed in fluid intake between sex, race/ethnicity, and levels of physical activity [17], the examination of 24 h urinary hydration measures is an important indicator of hydration status as it represents the behavioral and neuroendocrine responses that are regulated by fluid intake and normalizes any differences in body size, body composition, physical activity level, and solute load, which is difficult to control for in population-based studies. This study also represents the first step in further exploring racial and ethnic differences in daily hydration status; examination of sociocultural differences, social determinants of drinking behavior, and effects of daily fluid intake on health outcomes, particularly in populations with known health disparities is the strategic next step to further our understanding on this topic. 

Our study, however, is not without limitations. Utilizing a convenience sample of male and female college students from a large university in the southeastern part of the United States may not be generalizable to emerging adults from differing geographical, cultural, racial, and ethnic backgrounds. Furthermore, as we collected data throughout the academic year, we may not have been able to control for any acute changes in fluid intake behaviors that may have occurred in any one individual. Given the pilot nature of this work, we did not collect data related to daily fluid, or dietary intake and physical activity. Seeing as the former contributes to urine output across a 24 h period, prior evidence [47] suggests that a 24 h urine sample is a sufficient surrogate for daily fluid intake. Lastly, we must acknowledge that there is a potential that participants may not have provided a 24 h urine sample on each day; there may have been a missed void for a number of reasons. We feel the latter is of minimal concern considering our collection procedures required multiple and consecutive days of 24 h urine collection, thus we are confident that we were able to capture a representative average for each participant.

In conclusion, our findings show that a sample of non-Hispanic Black college students (particularly females) from the southeast part of the United States are inadequately hydrated compared to their non-Hispanic White counterparts. Furthermore, these findings show that Black male and female college students are not likely meeting recommended daily water intake volumes. Future research examining factors influencing the role of habitual fluid intake on health outcomes in populations with known health disparities such as obesity, cardiovascular disease, type II diabetes mellitus, etc. is needed to continue to develop proper prevention and management strategies to optimize long-term health.

## Figures and Tables

**Table 1:** Participant Demographics.

	Non-Hispanic White		Non-Hispanic Black	
	Male (*n* = 7)	Female (*n* = 3)	Male (*n* = 6)	Female (*n* = 19)
Age (y)	24 ± 4	24 ± 3	19 ± 1	19 ± 1
Height (cm)	173.9 ± 17.1	175.6 ± 10.9	169.1 ± 8.6	166.4 ± 11.4
Nude Body Mass (kg)	76.9 ± 10.7	72.5 ± 13.7	69.7 ± 9.8	69.6 ± 10.1
Body Fat (%)	17.7 ± 10.1	21.3 ± 9.2	20.3 ± 10.8	20.9 ± 10.2

**Table 2:** Weekday and weekend day 24 h urinary hydration measures.

	Non-Hispanic White		Non-Hispanic Black	
	Weekday	Weekend	Weekday	Weekend
Urine Volume (L)	2.13 ± 0.73	1.80 ± 0.57	0.84 ± 0.42	0.86 ± 0.47
Urine Osmolality (mOsm∙kg^−1^)	462 ± 192	498 ± 198	691 ± 250	768 ± 285
Urine Specific Gravity (AU)	1.013 ± 0.005	1.013 ± 0.006	1.019 ± 0.006	1.021 ± 0.007
Urine Color (AU)	3.1 ± 1.2	3.1 ± 1.2	4.1 ± 1.4	4.2 ± 1.5

**Table 3:** The 24 h urinary hydration measures between non-Hispanic White and non-Hispanic Black males and females.

	Non-Hispanic White		Non-Hispanic Black	
	Male (*n* = 7)	Female (*n* = 3)	Male (*n* = 6)	Female (*n* = 16)
Urine Volume (L)	1.96 ± 0.89	2.33 ± 0.87	0.89 ± 0.42	0.83 ± 0.44
Urine Osmolality (mOsm∙kg^−1^)	492 ± 195	389 ± 168	708 ± 252	719 ± 268
Urine Specific Gravity (AU)	1.013 ± 0.006	1.010 ± 0.004	1.020 ± 0.005	1.020 ± 0.007
Urine Color (AU)	3.2 ± 1.2	2.2 ± 0.9	4.4 ± 1.3	4.1 ± 1.4
